# Bridging Public Health Research and State-Level Policy: The Texas Research-to-Policy Collaboration Project

**DOI:** 10.5888/pcd21.240171

**Published:** 2024-10-07

**Authors:** Deanna M. Hoelscher, Alexandra van den Berg, Amelia Roebuck, Shelby Flores-Thorpe, Kathleen Manuel, Tiffni Menendez, Christine Jovanovic, Aliya Hussaini, John T. Menchaca, Elizabeth Long, D. Max Crowley, J. Taylor Scott

**Affiliations:** 1 Michael & Susan Dell Center for Healthy Living, Austin, Texas; 2 The University of Texas Health Science Center at Houston (UTHealth Houston) School of Public Health, Austin, Texas; 3 University of Illinois Cancer Center, Chicago, Illinois; 4 Michael & Susan Dell Foundation, West Lake Hills, Texas; 5 Emory University School of Medicine, Atlanta, Georgia; 6 Edna Bennett Pierce Prevention Research Center, The Pennsylvania State University, University Park, Pennsylvania

## Abstract

**Purpose and Objectives:**

Significant barriers to the implementation of evidence-based policy exist. Establishing an infrastructure and resources to support this process at the state level can accelerate the translation of research into practice. This study describes the adaptation and initial evaluation of the Texas Research-to-Policy Collaboration (TX RPC) Project, focusing on the adaptation process, legislative public health policy priorities, and baseline researcher policy knowledge and self-efficacy.

**Intervention Approach:**

The federal Research-to-Policy Collaboration (RPC) method was adapted to the Texas legislative process in 2020. Policymakers and public health researchers were recruited using direct outreach and referrals. Legislators or their aides were interviewed to determine health policy needs, which directed the development of legislator resources, webinars, and recruitment of additional public health researchers with specific expertise. Researchers were trained to facilitate communication with policymakers, and TX RPC Project staff facilitated legislator and researcher meetings to provide data and policy input.

**Evaluation Methods:**

Baseline surveys were completed with legislators to assess the use of health researchers in policy. Surveys were also administered before training to researchers assessing self-efficacy, knowledge, and training needs. Qualitative data from the legislator interviews were analyzed using inductive and deductive approaches. Quantitative survey data were analyzed using descriptive statistics for scales and individual survey items.

**Results:**

Legislative offices (n = 21) identified health care access, mental health, and health disparities as key health issues. Legislators reported that health data were important but did not actively involve researchers in legislation. Researchers (n = 73) reported that policy informed their work but had low engagement with legislators. Researcher training surveys indicated lower policy self-efficacy and knowledge and the need for additional training.

**Implications for Public Health:**

Adaptation of the RPC model for state-level health policy is feasible but necessitates logistical changes based on the unique legislative body. Researchers need training and resources to engage with policymakers.

**Summary**

**What is known on this topic?**

Although evidence-based policy can lead to better community health outcomes, public health researchers need support and resources to communicate their work to policymakers.

**What is added by this report?**

This project describes the state-level adaptation of a federal model that links researchers with policymakers to accelerate the implementation of evidence-based public health policy.

**What are the implications for public health practice?**

The Texas Research-to-Policy Collaboration Project determined emerging health priorities for the state legislative session and developed communication strategies and resources to link researchers with policymakers.

## Introduction

Although it is well accepted that nonmedical drivers of health or social determinants of health, such as housing, food insecurity, and transportation, exert significant effects on population health (1–3), recent scholars have begun to look further upstream at political determinants of health (4). Political determinants of health can be defined as the forces that reinforce or influence environmental or system-level factors that either exacerbate or attenuate health equity (4). These determinants, which include voting, government, and policy, are the forces that shape the environmental or systemic factors influencing health equity, either exacerbating disparities or contributing to their mitigation (4). Recognizing the critical interplay between civic participation and health, Healthy People 2030 has set a national health objective to increase the proportion of voting-age citizens who participate in elections (5). This acknowledgment underscores the importance of engaging with political determinants of health as a means to advance evidence-based health policies, highlighting a focus for health professionals aiming to improve health outcomes by bridging the gap between research findings and policy implementation.

Bridging the gap from health research to policy implementation remains a serious challenge, as seen during the COVID-19 pandemic. Researchers and policymakers have historically operated in distinct spheres with minimal collaboration (6). This separation is compounded by factors such as differing political structures, cultural beliefs and values, and the inherent trade-offs between costs and benefits, which introduces uncertainty into the process of advancing health policies at the population level. Further, the decision-making processes of researchers and policymakers are notably different and may hamper the connection and collaboration between the 2 groups (6,7). Researchers tend to engage in discrete, planned projects, whereas policymakers navigate a shorter, unplanned flow of tasks, often responding to immediate needs and unforeseen events. The difference in approach results in researchers needing time to collect or compile data to provide evidence-based recommendations, whereas policymakers must often make quick decisions, which are not always grounded in the empirical data that researchers value. Instead, policymakers may resort to rapidly acquired information, which may lack credibility. Recognizing and addressing these operational differences can enhance the translation of research into practice. Such efforts could substantially improve the effectiveness of public health policies and influence political determinants of health (8).

Even acknowledging the challenges and differences in operations between researchers and policymakers as a fundamental barrier, translation from research to policy is further complicated by additional and substantial barriers. Numerous studies have explored the mechanisms and challenges to better link research and policy (9–12). Caplan’s “two-communities” theory (13) from 1979 suggests that differing priorities, languages, and reward structures cause the gap between research and policy. Other barriers in translating research to policy include few direct interactions between policymakers and researchers, a lack of trust between policymakers and researchers, the perception that research is untimely or irrelevant, divergent communication styles, conflicting priorities, and budgetary pressures (8,10–12). This body of research emphasizes the importance of personal engagement and trusting relationships between researchers and policymakers to transfer research to policy effectively (14). Further, it underscores that these relationships are both timely and sensitive to the constraints policymakers face (15).

In response to these persistent barriers, recent initiatives have aimed to provide practical guidance for researchers to more effectively bridge the gap between research and policy. Haynes et al conducted a qualitative analysis of Australian civil servants (16) that identified 3 key attributes of researchers that policymakers particularly value: competence, integrity, and benevolence. Similarly, Oliver and Cairney’s (17) systematic review builds on this perspective by outlining a strategic framework for academic engagement in the policy process, emphasizing delivering high-quality research, fostering relationships with policymakers, and defining a clear stance as either a committed advocate or a neutral “honest broker.” Furthermore, there is growing consensus that a more sophisticated, bi-directional model enhances researchers’ ability to communicate findings, adapt projects to meet policy-relevant research questions, and understand organizational advocacy guidelines, while also encouraging policymakers to proactively engage with researchers ahead of legislative windows and offer political insights for selecting achievable policy-informed research objectives. This reciprocal relationship hinges on the establishment of trusted partnerships wherein researchers serve as honest brokers — an objective resource for navigating the complexities of policymaking (18,19).

Leveraging the insights from this body of research, the Research-to-Policy Collaboration (RPC) model was developed to facilitate personal researcher–policymaker relationships at the federal level and support the generation of timely and relevant evidence materials. A pilot study (7,20) of the RPC model was conducted, focusing on criminal justice policy efforts at the federal level. The aim of the study was to determine the effects on legislative–researcher connections and legislator and prevention scientist engagement, in addition to the cost-effectiveness of this approach. Ten Congressional offices participated in the pilot, which was conducted over 230 days. Results illustrated the efficacy and cost-effectiveness of this model for fostering personal connections between researchers and policymakers at the federal level. The study also highlighted that the most common legislative requests involved reviewing preventive intervention strategies (37%) and summarizing etiologic evidence (23%) (20).

The RPC model was developed to address systematic barriers and infrastructure needs that affect researchers’ engagement with policymakers to accelerate the implementation of evidence-based policy by using a 7-step process (21,22). Evaluation of this model shows a greater use of research evidence in legislative offices using this process and increased policy engagement among participating researchers (15,20). The RPC model comprises both capacity-building and collaboration components. More detailed descriptions of the RPC process can be found elsewhere (21), but an overview of the process is described below.

For capacity-building, Step 1 is assessing legislators’ policy goals and identifying policy champions on issues of interest. Once policy goals have been elucidated, researchers with relevant expertise are identified to form a resource network (Step 2). Step 3 involves matching policymakers with researchers who can provide relevant information or data to support legislative actions. Before engaging with legislators, researchers participate in training and ongoing technical assistance, providing context and skills to ensure effective communication between the legislator and the researchers (Step 4).

To build collaboration in policymaker–research matches, Step 5 of the RPC process entails rapid-response meetings, where legislative offices and researchers are brought together to discuss policy goals and develop a plan for relevant resource development and interactions. In Step 6, meetings are followed by initial strategic planning, which outlines goals, action steps, and timelines. Finally, Step 7 focuses on maintaining responsiveness to legislative interests and needs, which could include further involvement in the legislative process, such as testimony, research synthesis, or consultation. The success of this process depends on providing the infrastructure needed by researchers, most of whom are in academic institutions, to develop their knowledge transfer skills and provide needed resources for supporting the network and ongoing implementation (21).

## Purpose and Objectives

Given the success of the RPC model in a federal context, a natural and useful extension of this work is to explore its applicability and potential impact on public health policy within state-level legislative processes. With its distinctive legislative session, Texas presents an ideal case for this exploration and offers a unique opportunity to assess the model’s adaptability and effectiveness in a regional legislative environment. This article details the process of adapting a national framework of researcher–policymaker collaboration to the state level and presents initial evaluation data on the awareness and application of evidence-based policy practices among Texas researchers and policymakers, as well as lessons learned.

## Intervention Approach

The RPC model, designed for application at the federal level (20), was adapted for use within the Texas legislative process for the 2021 Legislative Session as the Texas Research-to-Policy Collaboration (TX RPC) Project (Table 1). This adaptation of the federal RPC evaluation and project tools was informed by prior research conducted with Texas legislators (23) and with input from the developers of the federal RPC model. Recommendations from the members of the project’s advisory committee, which included academic partners and health policy experts, and university governmental relations advisors, further informed the adaptation process. Key modifications were made to ensure the TX RPC Project aligned with the unique timelines and procedures of the Texas legislative cycle. An emphasis was placed on child health legislation and research, reflecting the state’s specific needs, potential areas of bipartisan agreement (24), and the investigators’ area of expertise. Beyond adapting the RPC model, the TX RPC Project expanded its outreach to include newsletters, webinars, and “lunch and learn” sessions at the Capitol, complemented by a legislative bill tracker to monitor relevant policy developments.

**Table 1:** Adaptation of the National Research-to-Policy Collaborative (RPC) to the Texas Research-to-Policy Collaborative (TX RPC)

Item	RPC (federal level)	Adaptation to TX RPC Project (state level)
Language	“Federal” survey language	Changed key words and/or questions to fit narrative of Texas legislators
Researcher policy training	Recorded with voice-over	Recorded live, uploaded on YouTube; held 2 refresher webinars
Policymakers	US federal legislators	Texas (state-level) legislators
Shared researcher site	Google docs	Basecamp
Rapid Response request form	Via email	Via Google Forms
Survey measurements	3 time series evaluations for researchers and legislators	Pre- and post-evaluation for researchers (survey) and legislators (interview); created researcher policy post-training survey; created COVID-19 legislator policy shift interview
Project timeline	Long: federal legislators are full-time	Short: Texas legislators are hybrid/part-time
Session period	1 Year, with each term having 2 sessions	Convene on odd years, 140 days of session every 2 years Agenda changes rapidly; leadership and interim charges
Researcher memberships	Network member	New network member categories: resource only; matched vs nonmatched
Group meetings, briefings, or presentations	Large collaborative/forum meeting	Project kick-off welcome reception at Texas State Capitol; multiple Lunch & Learn sessions at the Capitol, which included policy topic-specific presentations by researchers at the Capitol building with lunch or breakfast provided; webinars that provide policy topic information from a researcher
Facilitated collaborative meetings	In-person	Secure WebEx/Virtual meetings between researchers and legislators
Newsletter	Brief/topic specific	Evidence-based, featuring highlights and resources from TX RPC Project network members conducting COVID-19 and non-COVID-19 research and TX RPC Project partner events
Rapid Response products	Collecting and summarizing resources (eg, policy briefs); soliciting networks; briefings; hearings	Legislative Bill Tracker: since the 2013 Session, progress of selected health-related bills has been tracked during the legislative sessions and presented in an online format for legislators, researchers, and other advocates; collecting and summarizing resources (eg, policy briefs); soliciting networks; hearings

The Texas legislature operates under a schedule mandated by the state constitution, convening every odd-numbered year (eg, 2021, 2023) for 140 days. This biennial session typically spans from the beginning of January through the end of May. Governance is provided by the Governor and Lieutenant Governor, elected by the voters, and the Speaker of the House, elected by the House of Representatives. Texas uses a bicameral legislative system, with 150 House members and 31 Senators. House representatives serve 2-year terms, while Senators serve 4-year terms, with elections staggered so that approximately half the Senate is elected every 2 years. Despite the short session, the legislature typically has 7,000 or more bills filed.

Adapting the RPC methodology to Texas maintained all 7 steps of the model but required logistical and procedural modifications (Table 1). For example, the first 4 steps, which included assessing legislators’ goals, finding researchers with the appropriate expertise, matching legislators and researchers, and training researchers, were very similar to the federal RPC model. The short timeline of the Texas legislative session made it difficult to engage in many rapid-response meetings (Step 5) or to engage in significant strategic planning (Step 6), although we were able to maintain responsiveness to legislative needs (Step 7). Key format and procedural adaptations involved transitioning from federal to state-specific language and methods, which included updates in training protocols, use of shared sites for collaboration, and redesign of data request forms. Format and procedural adaptations included changes in language (federal to state) and methods (eg, training, shared sites, request forms). Modifying the RPC to fit the state legislature was complicated by the part-time availability of policymakers and their geographic dispersion across the state; thus, the RPC timeline was adjusted to accommodate a compressed, biennial legislative session. As a result, the evaluation design was altered to include a pre- and post-test survey and a different format for the researcher–legislator meetings, which included committees rather than one-on-one relationships. The unforeseen challenges of the COVID-19 pandemic and the need for physical distancing also led to a pivot to virtual meetings, enabling continued collaboration between policymakers and the TX RPC Project researchers and team members.

### Study population

#### Legislative offices

Since this study sought to understand the use of public health research in the Texas legislature, the sample was purposively recruited to represent legislators who lead policy efforts in child and public health. Elected officials in the Texas Senate and House of Representatives previously involved in public health policy, expressing an interest in the topic, appointed to relevant legislative committees such as health and human services, or members of select caucuses were invited to join the TX RPC Project in November 2019. Project staff sent a maximum of 4 recruitment emails to 68 legislative offices, with the goal of enrolling 20 legislative offices. Interested offices received a telephone call follow-up from study staff. A reception event at the state capitol was also held to inform these and other legislators about the study. Interested policymakers and their staff were scheduled for an in-person baseline survey and semi-structured policy identification needs-assessment interview. Baseline assessments were conducted in January, February, and May 2020 with 21 policymakers or their staff, who provided verbal consent to participate.

#### Researchers

Experts in health research from academic, nonacademic, and nonprofit settings in Texas were invited to join the TX RPC Project network of researchers in November 2019 based on their expertise and location in Texas. Project staff contacted 111 researchers by individual email but also recruited researchers through academic or state coalition listservs and an internal weekly newsletter. Up to 3 follow-up emails and phone calls were sent to interested researchers to enroll at least 50 researchers in the network. Researchers interested in participating completed the informed consent form, intake form, and baseline survey online using REDCap (Research Electronic Data Capture) software (25). Training to build capacity for engaging with legislators was offered in person and online to participating researchers, with a goal of training 20 to 30 researchers.

The Institutional Review Board of the University of Texas School of Public Health Committee for the Protection of Human Subjects (no. HSC-SPH-19–0539) reviewed the study and determined it to be exempt for both researchers and legislators.

### Collaborative partnerships

Researchers in the TX RPC Project Network were matched with participating legislative offices to meet to develop ongoing relationships and provide public health data and resources in preparation for the 2021 Texas Legislative Session. Researchers were matched to legislators based on 1) alignment with legislators’ district location, 2) expertise in reported public health policy priorities of the legislator, 3) availability to commit to a partnership, and 4) high level of engagement in the TX RPC Project Network. The project goal was to support 10 researcher–policymaker partnerships. Forty-five researchers were matched with 21 legislative offices to form collaborative partnerships. Additional researchers who were interested in the project but did not have the time for a legislator match were designated as “resource” members. These researchers served in an ad hoc role, providing expertise when needed for specific legislative requests or other activities (eg, webinars).

## Evaluation Methods

### Measures

The baseline survey for legislators or their aides was drawn from previous work that established the structural validity of scales used in this study (26). These surveys were conducted in person and assessed 1) use of research evidence (5 items); 2) perceived value of research evidence for policy work (6 items); 3) past and current interactions with researchers (8 items); 4) information sources (19 items); and 5) research training of office staff (4 items). For closed-ended questions, policymakers or their staff indicated agreement using a 5-point Likert scale (1 = not at all to 5 = all the time, or 1 = strongly disagree to 5 = strongly agree). Scales were developed for each construct by summing responses to the individual items.

The semi-structured policy identification needs assessment interview included semi-structured, open-ended questions to ask legislators or their designated staff about 1) policy priorities related to health, well-being, or wellness promotion, 2) strategies to strengthen the impact of policies, and 3) engaging researchers to support health policy efforts. Data from these interviews were used to help our research team prioritize health policy topics for briefs, webinars, and legislative bill tracking, as well as give us insight into each individual legislator’s need for information so that we could match legislators with researchers who had expertise in each health policy area.

The online researcher intake form to determine study eligibility collected self-reported demographic information (ie, contact information and organization affiliation), areas of research expertise, prior policy experiences, available time commitment, and preferences for online versus in-person training.

The researchers’ baseline survey was drawn from previous work that piloted these measures in the RPC pilot in Congress (7). The TX RPC Project surveys were conducted in an online format and assessed 1) prior policy experiences (1 item); 2) recent policy engagement (14 items); 3) policy-informed research activities (4 items); 4) perceived self-efficacy for engaging with public officials (10 items); 5) reported policy knowledge (7 items); and 6) training needs (9 items). For these closed-ended questions, response options varied; for questions asking about recent policy engagement, researchers responded on a 4-point categorical scale (1 = none to 4 = ≥7 times); for questions about policy-informed research activities, reported policy knowledge, and training needs, researchers responded on a 5-point Likert scale (1 = strongly disagree to 5 = strongly agree); and for questions regarding perceived self-efficacy engaging with public officials, researchers responded on a 4-point Likert scale (1 = not at all true to 4 = exactly true). Scales were developed for all constructs except prior policy experience by summing responses to individual items.

### Data analysis

All analyses were conducted using Microsoft Excel 2007/2016 for Windows XP and Stata software version 16.0 (StataCorp LLC). Descriptive statistics (including frequencies and percentages) were calculated for each question in the baseline assessments and for demographic information. For each set of questions, means, standard deviations, and Cronbach α were calculated for the scaled sum of items. Person mean imputation was performed for missing data, where at least 1 item in the scale was nonmissing, to minimize bias introduced by simple case deletion (27). For the researcher training evaluation, mean and standard deviation were calculated for each question and scale. Policy priorities were categorized by health topic at baseline. Semi-structured interview notes were entered into a database, and three research team members performed content analysis to identify relevant themes using standard inductive qualitative methods (28,29).

## Results

Legislators (n = 21) included both senators and representatives, with a majority (57.1%) from the Texas House of Representatives; most were female (61.9%). Almost all (90.5%) legislators participated in re-election campaigns in November 2020. Eighty-three researchers enrolled in the TX RPC Project Network, and 59 completed the policy capacity training. Researchers were predominantly female (66.3%) and academics with a university affiliation (89.1%).

Interviews were conducted with legislators and their staff, including legislative directors, chiefs of staff, legislative assistants and aides, policy analysts, an education specialist, and a district director. The most frequently mentioned health policy priority (Table 2) was health care access/Medicare/Medicaid, which 76.2% of legislators identified as a significant area of concern. Health policy topics mentioned by 40% or more of the respondents included mental health, health disparities, vaping/e-cigarettes, and maternal and child health. Other health policy priorities were Supplemental Nutrition Assistance Program (SNAP)/food insecurity, school nutrition, immunizations, disabilities, and violence prevention (Table 2). Because most legislative interviews were conducted in early 2020, COVID-19–related health policy was rarely mentioned.

**Table 2:** Healthy Policy Priority Topics Identified in Initial Texas Research-to-Policy Collaborative Project Legislator Interviews (n = 21)

Health policy priority topic	Frequency (%)
Health care access/Medicare/Medicaid	16 (76.2)
Mental health	13 (61.9)
Health disparities	12 (57.1)
Vaping/e-cigarettes	11 (52.4)
Maternal and child health	11 (52.4)
SNAP/food insecurity	7 (33.3)
Nutrition	6 (28.6)
Immunizations	6 (28.6)
People with disabilities	5 (23.8)
Violence prevention	5 (23.8)
Chronic disease	4 (19.0)
Substance use prevention, injury prevention, sexual health, environmental health, health care workforce development	3 (14.3)
Border health, dental/oral health, women’s health, physical activity, nonhealth-related issues, other	2 (9.5)
Screen time/sedentary behavior, healthy aging	1 (4.8)

Abbreviation: SNAP, Supplemental Nutrition Assistance Program.

Baseline surveys were collected from 73 researchers and 21 legislators (Table 3). Most researchers indicated little prior engagement with policymakers, especially during the policy creation process. In general, researchers tended to agree that policy informed their work but also reported moderate levels of policy-related self-efficacy and policy knowledge. Most researchers reported that they needed more training regarding policy-related training needs (Table 3).

**Table 3:** Baseline Survey Responses from TX RPC Project Researchers (n = 73) and Legislators (n = 21)

Baseline Survey Assessment	Mean (SD)
**Researchers**	
**Prior engagement with policymakers (1 = none; 4 = ≥7 times):**	
Interpreted science for policymakers	1.95 (1.00)
Integrated science into decision-making	2.08 (1.11)
Actively taken a position on an issue based on scientific results	1.96 (1.09)
Acted as a decision-maker with regard to a policy	1.44 (0.85)
Proactively contacted policymakers to talk about research related to policy issues^a^	1.89 (1.07)
Was contacted by policymakers about research or policy issues	1.95 (1.03)
Shared research articles, reports, or materials with a policymaker	2.26 (1.11)
Scale	13.52 (5.42)
Cronbach α	0.87
**Engaged with policymakers when they were (1 = none; 4 = ≥7 times):**	
Setting their agenda^a^	1.63 (0.91)
Developing a policy or program	1.85 (0.98)
Considering policy or program implementation	1.92 (1.02)
Evaluating a policy or program’s effectiveness or impact^a^	1.68 (0.98)
Deciding about the content or direction of a policy or program^a^	1.76 (0.88)
Persuading others to a point of view or course of action	1.89 (1.03)
Required or expected to use research	1.86 (1.10)
Scale	12.60 (5.77)
Cronbach α	0.93
**Policy-informed research (1 = strongly disagree; 5 = strongly agree); *My work and/or research . . .***	
Is informed by major policy issues	3.99 (1.05)
Incorporates advice or information from policymakers and information	3.44 (1.04)
Considers the priorities of policymakers	3.68 (1.01)
Considers the needs of policymakers	3.64 (1.05)
Scale	14.75 (3.48)
Cronbach α	0.86
**Policy-related self-efficacy (1 = not at all true; 4 = exactly true); *In conversations with policymakers’ staff . . .***	
I can discuss solving difficult problems^a^	3.13 (0.74)
If staff oppose me, I can find means to negotiate our different viewpoints^a^	2.97 (0.77)
It is easy for me to stick to my aims and accomplish my goals^a^	3.15 (0.72)
I am confident that I could deal efficiently with unexpected issues that come up^a^	2.97 (0.77)
I am resourceful during meetings, which allows me to handle unforeseen situations^a^	3.03 (0.86)
I can discuss most problems without eliciting conflict if I invest the necessary effort^a^	3.34 (0.70)
I can remain calm when facing difficulties because I can rely on my coping abilities^a^	3.51 (0.63)
When staff confront me with a problem, I can usually find several solutions^a^	3.07 (0.82)
If I encounter conflict during discussion, I can usually think of a solution^a^	3.10 (0.81)
I can usually handle whatever comes my way during conversations^a^	3.15 (0.78)
Scale	31.07 (6.95)
Cronbach α	0.96
**Reported policy knowledge (1 = strongly disagree; 5 = strongly agree); *I understand . . .***	
The multiple factors that policymakers must consider when making decisions^a^	3.93 (1.12)
The primary information sources policymakers use for decision-making^a^	3.36 (1.10)
How policymakers’ timeframe for action differs from researchers’^a^	3.85 (1.11)
The ways in which policymakers define evidence^a^	3.28 (1.12)
Common perceptions of researchers that hinder developing trusting relationships with policymakers^a^	3.33 (1.13)
How to make contact with legislative offices^a^	3.49 (1.14)
Ways I can seek to support legislative offices^a^	3.11 (1.12)
Scale	24.35 (6.38)
Cronbach α	0.92
**Training needs (1 = strongly disagree; 5 = strongly agree); *I need more training for . . .***	
Communicating with policymakers and staff^a^	3.80 (1.06)
Understanding the legislative process and where researchers can play a role^a^	3.86 (1.16)
How to work with legislative offices^a^	3.84 (1.03)
Best practices for synthesizing literature^a^	3.01 (1.20)
Responding to needs of legislative offices^a^	3.86 (1.00)
Regulations on lobbying and advocacy^a^	3.93 (1.07)
Maintaining a working relationship with legislative offices^a^	3.97 (1.02)
Engaging with advocacy groups^a^	3.36 (1.22)
Responding or engaging with media^a^	3.55 (1.18)
Scale	33.32 (7.78)
Cronbach α	0.92
**Legislators**	
**In the past legislative session, used research to (1 = not at all; 5 = all the time):**	
Set a policy agenda	4.19 (0.68)
Develop a specific policy	4.38 (0.74)
Consider how an agency might implement a policy	3.86 (0.91)
Persuade others to a point of view or course of action	4.24 (0.77)
Mobilize support for important issues	4.19 (0.87)
Scale	20.86 (2.74)
Cronbach α	0.72
**Valuable to use research in work to (1 = strongly disagree; 5 = strongly agree):**	
Identify issues that require a policy or program response	4.67 (0.48)
Decide about specific content	4.52 (0.51)
Persuade others to a point of view or course of action^b^	4.60 (0.60)
Craft legislation	4.48 (0.51)
Scale	18.25 (1.43)
Cronbach α	0.62
**In general, find that research is (1 = strongly disagree; 5 = strongly agree):**	
Useful	4.86 (0.36)
Underutilized by policymakers	4.05 (1.02)
**In the past legislative session, when developing policies or bills, interacted with researchers by (1 = not at all; 5 = all the time):**	
Attending forums (eg, conference, briefing, webinar) to hear about research findings^b^	3.50 (0.83)
Working with researchers to identify policy direction or priorities	3.48 (0.93)
Working with researchers to identify research direction or priorities	2.33 (1.02)
Inviting researchers to be an active contributor to policy development	3.43 (0.93)
Collaborating with researchers to interpret research findings^b^	2.95 (0.89)
Calling on researchers for advice on bill development for proposed legislation	3.57 (1.03)
Calling on researchers to testify at a hearing^b^	3.85 (1.04)
Obtaining data from researchers	3.62 (0.97)
Scale	26.77 (5.77)
Cronbach α	0.87
**Rely on each of the following to obtain policy-related information (1 = not at all; 5 = all the time):**	
Academic journals or research reports about an issue	4.00 (1.22)
People who are involved with a program or policy we are dealing with	4.43 (0.68)
Researcher from a local college or university	3.14 (1.01)
Someone who presented at a briefing, research forum, conference, or training workshop	3.43 (0.81)
Web-based clearinghouses	3.62 (0.97)
Intermediary organizations like Texas Medical Association	4.19 (0.75)
A consultant (eg, a lobbyist or nonpartisan advisor) to obtain it for me	3.81 (0.81)
Staff members from federal or state government agencies	4.10 (0.77)
Non-profit organizations/foundations	4.24 (0.62)
Conferences or training workshops	3.14 (0.96)
Meeting with professionals in the district or state	3.81 (0.81)
From constituents	3.67 (1.15)
National data	3.71 (0.56)
Texas data	4.33 (0.48)
Local data	3.90 (0.83)
Texas Child Health Status — Healthy Children, Healthy State report series	2.19 (0.98)
Traditional media (news clips, cable news, etc.)	3.43 (0.93)
Legislative leadership offices (Lt. Gov, Speaker, committee offices)	3.29 (1.27)
Information from committee testimony	4.00 (0.84)
Scale	70.43 (7.98)
Cronbach α	0.81

Abbreviations: TX RPC, Texas Research-to-Policy Collaboration; SD, standard deviation.

a Missing, researchers: proactively contacted policymakers to talk about research related to policy issues, n = 72; setting their agenda, n = 72; evaluating a policy or program’s effectiveness or impact, n = 72; deciding about the content or direction of a policy or program, n = 72; I can discuss solving difficult problems, n = 70; if staff oppose me, I can find means to negotiate our different viewpoints, n = 69; it is easy for me to stick to my aims and accomplish my goals, n = 67; I am confident that I could deal efficiently with unexpected issues that come up, n = 69; I am resourceful during meetings, which allows me to handle unforeseen situations, n = 68; I can discuss most problems without eliciting conflict if I invest the necessary effort, n = 68; I can remain calm when facing difficulties because I can rely on my coping abilities, n = 69; when staff confront me with a problem, I can usually find several solutions, n = 68; if I encounter conflict during discussion, I can usually think of a solution, n = 68; I can usually handle whatever comes my way during conversations, n = 67; the multiple factors that policymakers must consider when making decisions, n = 72; the primary information sources policymakers use for decision-making, n = 72; how policymakers’ timeframe for action differs from researchers, n = 72; the ways in which policymakers define evidence, n = 72; common perceptions of researchers that hinder developing trusting relationships with policymakers, n = 72; how to make contact with legislative offices, n = 71; ways I can seek to support legislative offices, n = 70; communicating with policymakers and staff, n = 70; understanding the legislative process and where researchers can play a role, n = 71; how to work with legislative offices, n = 70; best practices for synthesizing literature, n = 72; responding to needs of legislative offices, n = 70; regulations on lobbying and advocacy, n = 71; maintaining a working relationship with legislative offices, n = 70; engaging with advocacy groups, n = 69; responding or engaging with media, n = 71).

b Missing, legislators: persuade others to a point of view or course of action, n = 20; attending forums (eg, conference, briefing, webinar) to hear about research findings, n = 20; collaborating with researchers to interpret research findings, n = 20; calling upon researchers to testify at a hearing, n = 20).

Legislative offices generally reported a high use of research in policy development, and many strongly agreed that it was valuable to use research in their work. Despite these views, legislative offices reported few interactions with researchers, especially working with researchers to identify research direction or priorities (eg, informing researcher work). Legislative offices often obtained policy-related information from people involved with the policy or program, Texas data, and nonprofit organizations or foundations; however, fewer legislative offices reported obtaining information from researchers, conferences, or program-specific materials (Table 3).

## Implications for Public Health

Adaptation of the RPC to a state-level government is feasible but must include changes in logistics that depend on the unique structures and practices of the state legislative body. Health policy priorities identified at the state level by legislative offices included mental health, vaping/e-cigarette use, health disparities, health care access, and food insecurity, all important public health issues. Baseline survey data showed that researchers do not frequently engage with legislative offices in a structured manner across the policy development spectrum and that researchers require training in these skills. Legislative officials realize the value of research, especially state-level data, but do not often engage with researchers. Thus, there is a need for a coordinating organization and process to develop and maintain trusted relationships between researchers and legislative offices.

The adaptation of the RPC process to Texas posed unique issues. For example, Texas is 1 of only 4 states with a legislature that meets on a biennial basis (30). This compressed schedule leads to policy development before the actual session and often before the election. Most Texas legislators tend to reside in the state capitol for the 140-day term and then return to their districts and a primary job. In terms of establishing partnerships between policymakers and researchers, this schedule leads to fewer opportunities to interact with policymakers, the necessity of interacting with policymakers in their home districts rather than in the Capitol, and limited time to develop new legislation. The short session also required proactive steps to develop a library of potential resource materials ahead of actual requests based on input from the project advisory committee.

Conducting this work during a pandemic also presented several challenges but additional opportunities. Originally, in-person meetings between researchers and legislators were proposed, which would have necessitated travel expenses for researchers across the state. Since the COVID-19 pandemic led to social distancing, many offices were not at the Capitol during the interim period (2020), and researcher–legislative office meetings were pivoted to an online secure video forum using WebEx. Although the project was not able to arrange face-to-face meetings, the video format provided project cost savings and easier scheduling. The pandemic also resulted in several requests for COVID-19 and vaccine-related data and information, which became a major focus of the TX RPC Project.

As with other studies (15,20), our work indicated that researchers generally do not actively engage with policymakers and need additional training, especially in facilitating communication and building trusted relationships with legislators (6,9,31). The RPC project provided training, update meetings, communications, support from the research staff, and resources for both researchers and legislators. This support is necessary, as policy-related activities, which fall under knowledge translation or community-engaged research, often do not count toward the tenure and promotion metrics valued in traditional academic environments (9,21,32). Recent work has called for better inclusion of these activities as academic metrics, in addition to bibliographic output and funding, but universities have not widely adopted this practice (32,33).

Although legislative offices reported using research in health policy development, few reported connecting directly with academics and researchers. Consistent with prior research, potential reasons for limited contact between legislative offices and researchers include inadequate translation of complex research into practical recommendations (34), infrequent interactions between policymakers and legislative offices, and differences in culture (10,11). Training is necessary to overcome some of these communication barriers (31). Building trusted relationships between researchers and legislators facilitates advancing evidence-based policy, providing further evidence for the TX RPC Project approach (12). A recent adaptation of the RPC model, the SciComm Optimizer for Policy Engagement (SCOPE), connected researchers and state legislators using email and was evaluated by using a randomized controlled trial. Results showed that legislators who received the intervention were more likely to use research evidence in their social media posts (35). The TX RPC Project model included more engagement and outreach than the SCOPE process, which might be necessary for a short legislative session like that in Texas. Thus, models for connecting researchers and state legislators can accelerate the knowledge transfer of research into public health action, but further evaluation methods should be used to determine effectiveness.

The TX RPC Project model sought to accelerate the adoption and reach of evidence-based public health policy (36) at the state level. The TX RPC Project was the first adaptation of the federal RPC process in which the model developers did not oversee its implementation. Thus, the TX RPC Project provides evidence of scalability and institutionalization of the RPC process in a different setting, suggesting that implementation science approaches can be applied to health policy work (36).

Although the TX RPC Project has several strengths, including building on an existing evidence-based model, current governmental and legislative relationships, and evaluation of the process, there are several limitations. Twenty-one legislators participated in the project, so their views may not reflect the dominant views in the Texas legislature. Alternatively, these legislators might represent the subset interested in advancing public health policy. The participating legislators were identified for their interest in health policy but not for their views on other policy domains. Ideally, every legislator in the Texas legislature would be recruited, but given the short session and limited resources, we chose to prioritize those legislators who were most likely to file and advance health policy legislation. The Texas RPC newsletters were sent to all Texas legislators, and a request form was included that invited legislative offices to contact us for any information related to health policy. Additionally, many of the outcomes of this study are largely based on self-reported process-oriented data. Still, this study demonstrates the utility of the RPC model in a state environment and provides an in-depth evaluation of how Texas legislators view public health researchers as experts in developing effective health policy at the state level.

The TX RPC Project illustrates some of the benefits and challenges of linking researchers to policymakers using a model that has been successful at the federal level. With the past few years of federal Congressional gridlock, state legislators are a promising means of innovating health policy (37). Thus, further work at enhancing strategies that promote evidence-based health policy that addresses the political determinants of health (4) at the state level can lead to improved health outcomes that can, in turn, be used as exemplars for other states and the nation.
